# Correlation Between Intratumoral NETs and Neutrophil-to-Lymphocyte Ratio in Gastric Cancer

**DOI:** 10.7759/cureus.95894

**Published:** 2025-11-01

**Authors:** Masahiro Takeuchi, Chise Shiraishi, Yurika Fukudome, Masahiko Onoda, Michinori Iwamura, Toshihiro Inokuchi, Kazuaki Kawano, Tomoe Katoh, Keiji Hirata, Akira Furutani

**Affiliations:** 1 Department of Surgery, Yamaguchi Rosai Hospital, Sanyo-Onoda, JPN; 2 Department of Surgery 1, University of Occupational and Environmental Health, Kitakyushu, JPN

**Keywords:** gastric cancer, neutrophil extracellular traps, neutrophil lymphocyte ratio, prognosis, surgery

## Abstract

Purpose: Systemic inflammation is increasingly recognized for its impact on the progression of cancer and patient prognosis. Among relevant biomarkers, the neutrophil-to-lymphocyte ratio (NLR) has emerged as a prognostic factor for multiple malignancies. However, the clinical implications of both the circulating NLR and intratumoral neutrophil extracellular traps (NETs) in gastric cancer remain unclear. This study aimed to explore the association between preoperative NLR and the presence of NETs in tumor specimens and to evaluate their prognostic relevance.

Methods: A retrospective analysis was conducted on 52 patients who underwent curative surgery for gastric cancer between 2017 and 2018. Pre-surgical NLR values were derived from routine blood tests, and patients were stratified into two groups: NLR ≥3.5 and NLR <3.5. Correlations between tissue-based NETs and systemic NLR, as well as overall survival outcomes, were assessed.

Results: Patients in the high NLR group exhibited significantly reduced median survival compared to those in the low NLR group (2.60 vs. 3.98 years, p = 0.014). A moderate positive correlation was observed between the NLR and NET density in tumor tissues (r = 0.507; 95% CI: 0.2714-0.685; p = 0.0001).

Conclusion: An elevated NLR is associated with poor survival outcomes in patients with gastric cancer and is positively correlated with NET formation in the tumor environment. These findings suggest that the NLR, along with tissue NETs, could serve as an accessible prognostic tool in clinical practice.

## Introduction

In recent years, neutrophils have been increasingly recognized for playing a pivotal role in cancer progression. These cells exhibit remarkable phenotypic plasticity in response to various micro-environmental and external stimuli. Notably, the discovery of neutrophil extracellular traps (NETs), a novel functional form of neutrophils, has led to a surge in research on their involvement in cancer. Emerging evidence suggests that NETs may contribute to tumor development and metastasis and are being explored as potential biomarkers and therapeutic targets [1,2].

The neutrophil-to-lymphocyte ratio (NLR), which reflects the host systemic inflammatory response, is a simple yet highly useful prognostic indicator for gastric cancer that can be readily assessed through routine blood tests [3]. Despite the growing interest in both NETs and the NLR in the context of cancer, few studies have comprehensively evaluated the relationship between tissue-localized NETs and systemic inflammatory markers, such as the NLR. In this study, we aimed to evaluate the clinical significance of tissue NETs and the systemic NLR in patients with gastric cancer and to investigate the potential role of NETs in the context of systemic inflammation and tumor progression. We herein report that serum biomarkers, such as the NLR, are correlated with NETs in cancer tissues.

## Materials and methods

Patients and clinical data

**Figure 1:**
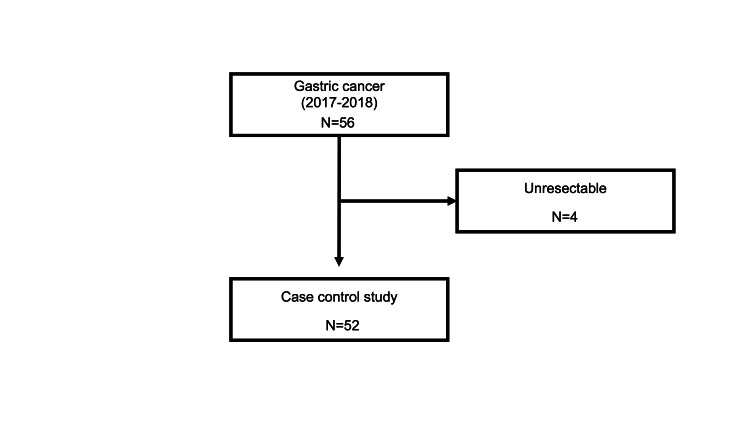
Flowchart of this study.

This retrospective cohort study included patients diagnosed with gastric cancer who underwent surgical exploration at Yamaguchi Rosai Hospital between 2017 and 2018 (Figure 1). The primary endpoint of this investigation was cancer-specific survival. Clinical and laboratory data were obtained, including patient demographics (age and sex), inflammatory markers such as C-reactive protein (CRP), tumor markers including carcinoembryonic antigen (CEA) and carbohydrate antigen 19-9 (CA19-9), and pathological staging based on the seventh edition of the American Joint Committee on Cancer (AJCC) guidelines for gastric cancer. The presence of comorbidities, such as diabetes mellitus, was also noted.

Preoperative complete blood counts were systematically collected, including hemoglobin levels, platelet counts, and differential white blood cell counts (neutrophils, lymphocytes, and monocytes). Patients with inadequate follow-up data were excluded from the final analysis. At the time of study enrollment, both clinical information and survival outcomes were retrieved from institutional medical records. Overall survival (OS) was defined as the interval from the date of the diagnosis until death or the last known follow-up examination.

This study was approved by the Institutional Review Board of Yamaguchi Rosai Hospital (approval number: Yro-ri-18) and adhered to the ethical principles outlined in the Declaration of Helsinki (1964) and subsequent revisions.

Immunohistochemical evaluation of citrullinated histone H3

Primary tumor tissues preserved in formalin and embedded in paraffin were sectioned at a thickness of 4 μm. The sections were first stained with hematoxylin and eosin (H&E) to assess their general morphology. For immunohistochemical detection, antigen retrieval was performed by heating sections in 1 mM EDTA buffer (pH 8.0). Subsequently, to detect the neutrophils that were transformed into NETs, the slides were incubated for 1 h at room temperature with a rabbit monoclonal antibody targeting citrullinated histone H3 (RM1001; Abcam Japan) at a dilution of 1:200. Visualization was performed using the Simple Stain MAX PO (M) kit, with diaminobenzidine tetrahydrochloride (DAB) as the chromogen (Nichirei Biosciences, Tokyo, Japan).

The entire tumor area on each slide was examined at 20× magnification to assess neutrophil and NET infiltration. Regions showing clear positive staining were identified, and three representative fields were randomly chosen for quantitative analysis. The number of positively stained cells in these fields was counted manually. The percentage of NETs was calculated by dividing the number of citrullinated histone H3-stained neutrophils by the total number of neutrophils. All evaluations were conducted by a blinded observer with no knowledge of the patients’ clinical outcomes or other experimental data.

Statistical methods

All statistical analyses were performed using JMP (ver. 13.0; SAS Inc., Cary, NC, USA). Depending on the data distribution characteristics, group comparisons were made using either Student’s t-test or the non-parametric Mann-Whitney U test. Associations between continuous variables were examined using a linear regression analysis. Receiver operating characteristic (ROC) curve analyses were used to determine the optimal cut-off values for continuous parameters. OS was analyzed using the Kaplan-Meier method, and intergroup differences were evaluated using the log-rank test. Statistical significance was defined as a two-tailed p-value of < 0.05.

## Results

Patient characteristics

Figure 1 and Table 1 present an overview of the clinical features of the patient cohort. A total of 56 were initially diagnosed with gastric cancer. Among them, four cases were excluded because they were unresectable. A total of 52 patients underwent curative surgery were included in the analysis.

**Table 1:** Patient characteristics. The data represent the number for males and females (52). (%) = relative frequency. NLR, neutrophil-to-lymphocyte ratio; BMI, body mass index; LN, lymph node; CRP, C-reactive protein; CEA, carcinoembryonic antigen; CA19-9, carbohydrate antigen 19-9; df, degree of freedom; χ2, chi-square. *p<0.05.

	Low NLR	High NLR	df = 1, χ2	p value
Patients (%)	36 (100)	16 (100)		
Gender			2.17	0.141
Men	22 (61.1)	13 (81.3)		
Women	14 (38.9)	3 (18.7)		
Age (year)			2.67	0.102
<73	20 (55.6)	5 (31.3)		
≥73	16 (44.4)	11 (68.7)		
Neoadjuvant chemotherapy			2.40	0.121
(-)	36 (100)	15 (93.7)		
(+)	0 (0)	1 (6.3)		
Smoking			0.43	0.513
(-)	17 (47.2)	6( 37.5)		
(+)	19 (52.8)	10 (62.5)		
BMI			1.17	0.280
<25	30 (88.3)	15 (93.7)		
≥25	6 (16.7)	1 (6.3		
Diabetes			2.03	0.155
(-)	31 (86.1)	11 (68.7)		
(+)	5 (13.9)	5 (31.3)		
Differentiation			4.83	0.028*
tub1 / tub2 / pap	29 (80.6)	8 (50.0)		
por / sig	7 (19.4)	8 (50.0)		
Invasion			1.94	0.232
	21 (58.3)	6 (37.5)		
≥MP	15 (41.7)	10 (62.5)		
Lymph node metastasis			0.62	0.551
(-)	20 (55.6)	7 (43.7)		
(+)	16 (44.4)	9 (56.3)		
CRP (mg/dL)			7.34	0.007*
<0.5	32 (88.9)	8 (53.3)		
≥0.5	4 (11.1)	7 (46.7)		
Hemoglobin (g/dL)			3.87	0.049*
<11	8 (22.2)	8 (50.0)		
≥11	28 (77.8)	8 (50.0)		
Platelet (×10^4^)			1.84	0.175
<20	16 (44.4)	4 (25.0)		
≥20	20 (55.6)	12 (75.0)		
Neutrophils (%)			51.64	<0.0001*
<70	34 (94.4)	0 (0.00)		
≥70	2 (5.6)	16 (100)		
Lymphocytes (%)			55.00	<0.0001*
<20	0 (0.00)	15 (93.7)		
≥20	36 (100)	1 (6.3)		
Monocytes (%)			3.80	0.059
<5.5	10 (27.8)	9 (56.3)		
≥5.5	26 (72.2)	7 (43.7)		
CEA (U/mL)			0.07	0.790
<5.0	25 (71.4)	12 (75.0)		
≥5.0	10 (28.6)	4 (25.0)		
CA19-9 (U/mL)			0.02	0.892
<37	31 (86.1)	14 (87.5)		
≥37	5 (13.9)	2 (12.5)		

Based on the cutoff value obtained using the ROC curve, the population was divided into high (≥3.5) and low (<3.5) NLR groups for analysis. Elevated CRP levels (p = 0.007) and a higher incidence of anemia (p = 0.049) were observed in the high NLR group. The high NLR group had a high proportion of undifferentiations (p = 0.028).

Analysis of survival

**Figure 2:**
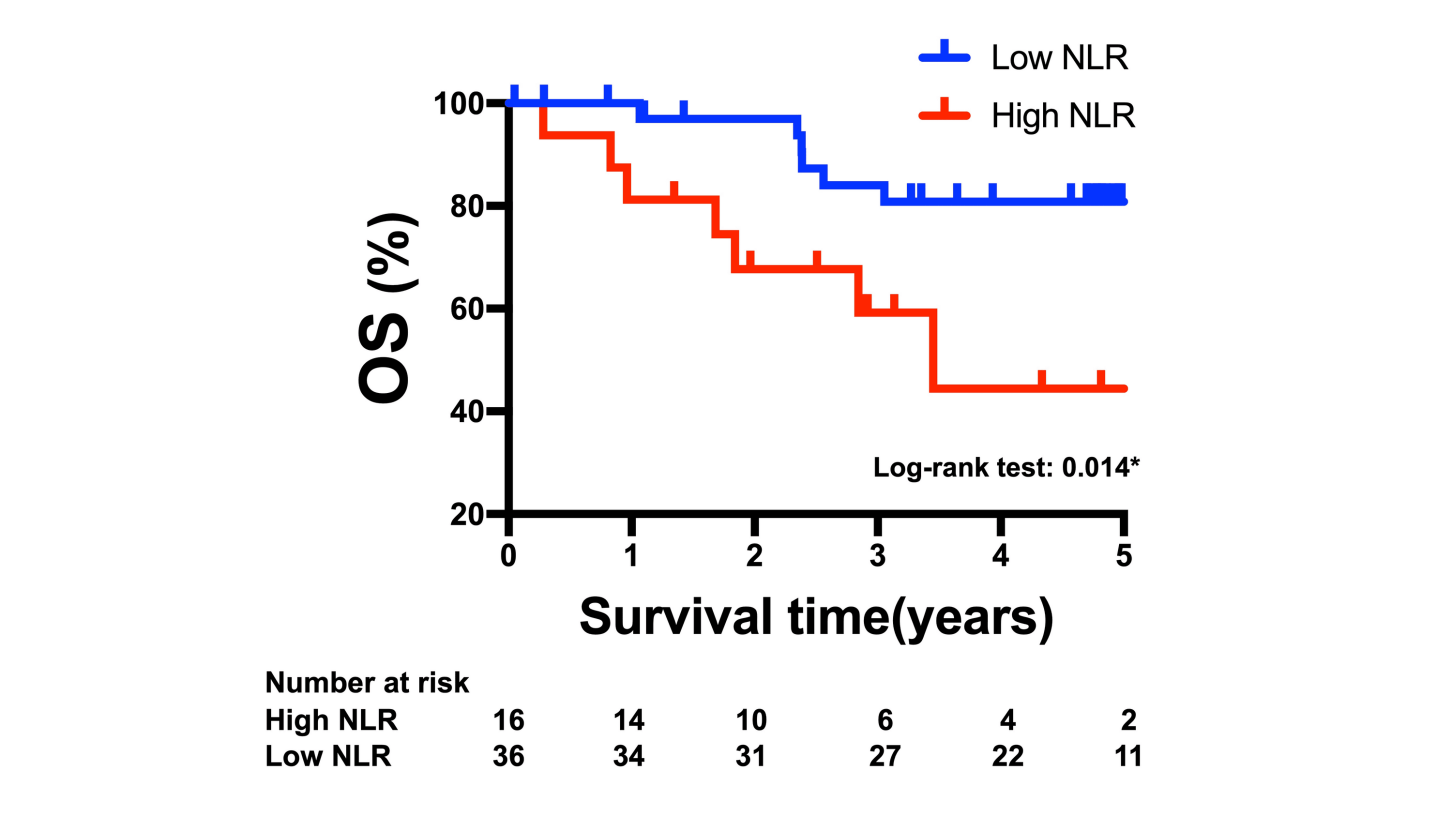
Kaplan-Meier curves of cancer-specific survival in patients according to the NLR. OS, overall survival; NLR, neutrophil-to-lymphocyte ratio. Data represent *p<0.05, compared to high NLR.

OS was significantly worse in patients with a high NLR (Figure 2, p = 0.014 by the log-rank test).

Correlations between the NLR and tissue NETs

**Figure 3:**
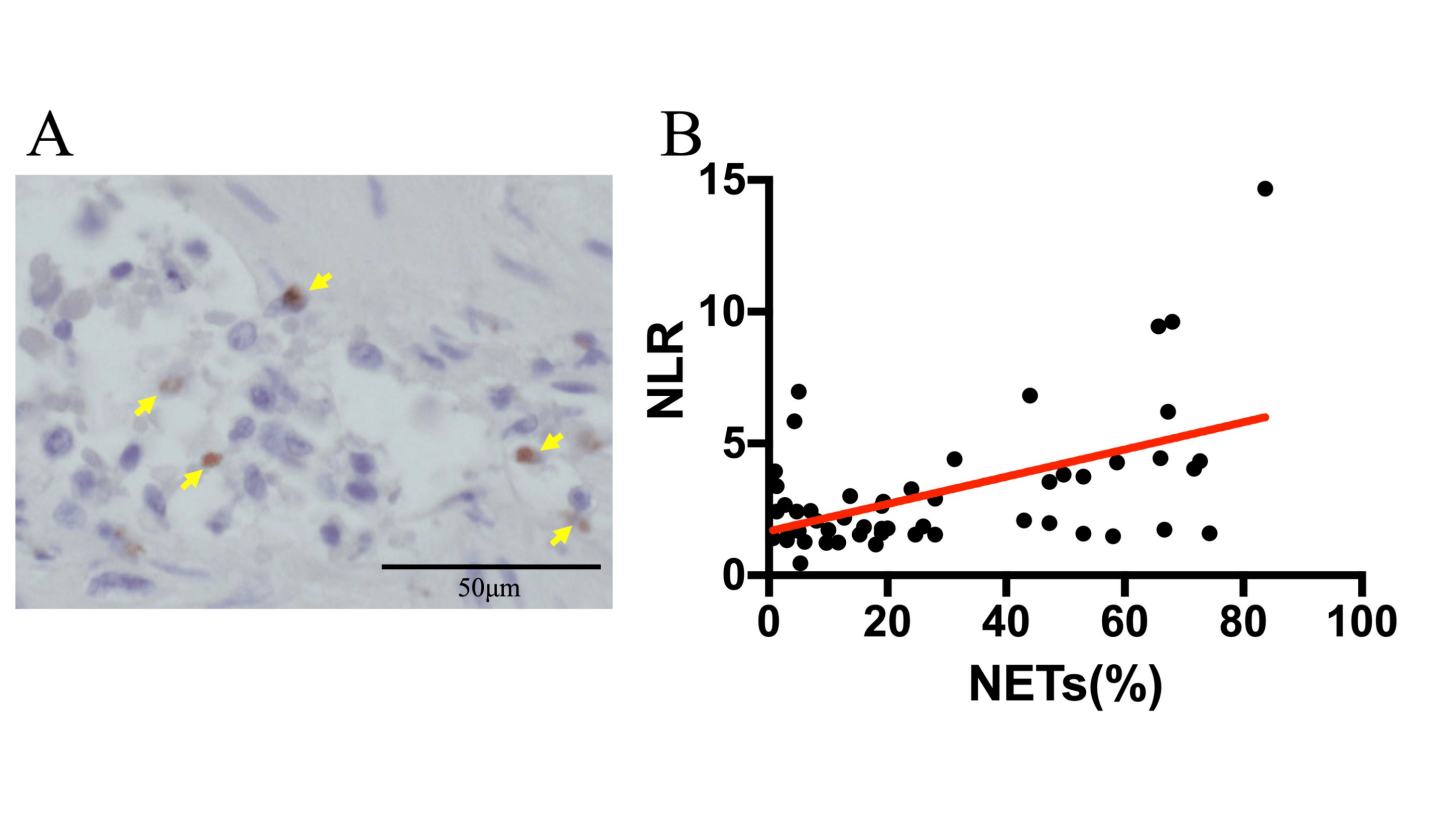
Immunohistochemical detection of NETs in gastric cancer. (A) Representative case. Stained by brown: citrullinated Histone 3 (yellow arrow). (B) Correlation of the NLR and NETs in cancer tissue. NLR, neutrophil-to-lymphocyte ratio; NETs, neutrophil extracellular traps.

Sections from 52 specimens were stained with HE and examined for the presence of neutrophils. A representative case is shown in Figure 3A. NETs were identified by immunostaining citrullinated histone H3. We detected a correlation between the NLR and tumor tissue NETs (Figure 3B, r = 0.507, 95%CI = 0.2714-0.685, p = 0.0001).

## Discussion

Neutrophils play a critical role in host defense by migrating to sites of infection where they become activated. Activated neutrophils phagocytose pathogens and generate reactive oxygen species (ROS) to kill the ingested microbes. A groundbreaking discovery by Brinkmann et al. [4] revealed that unlike other immune cells, activated neutrophils release nuclear chromatin into the extracellular space. These chromatin fibers, termed NETs, form web-like structures that entrap pathogens at the site of infection. Entrapped pathogens are more readily phagocytosed by neutrophils and macrophages, and NETs possess direct bactericidal activity [5].

In addition to infection control, NETs have been shown to contribute to thrombosis by trapping and activating platelets [6], cancer metastasis [7], and the pathogenesis of autoimmune diseases such as systemic lupus erythematosus [8], among others. These findings have expanded the significance of NETs from basic cell biology to clinical medicine, where they are now considered important contributors to various disease processes, including malignancy.

Neutrophils and lymphocytes, both essential components of the immune system, are classified as inflammatory cells that accumulate at sites of inflammation. Neutrophils are primarily involved in acute inflammation, while lymphocytes play a central role in chronic inflammation. Consequently, the balance between these two cell types, represented by the NLR, serves as a marker not only of immune status but also of systemic inflammation. An elevated NLR reflects a heightened inflammatory state and is associated with advanced malignancy [9]. In patients with cancer, a high NLR is thought to reflect both increased tumor-associated inflammation and reduced anti-tumor immunity, such as diminished tumor-infiltrating lymphocytes. Several studies have reported that a high NLR is associated with a poor prognosis in various malignancies, including gastric cancer [10].

Recent studies have further highlighted the pro-tumorigenic role of NETs in cancer biology. For instance, NETs have been implicated in the formation of a pro-metastatic niche by entrapping circulating tumor cells, facilitating distant metastasis [1,7,11]. Demers and Wagner [11] emphasized that NETs not only promote tumor cell adhesion and extravasation but also modulate the tumor immune microenvironment.

Neutrophils and lymphocytes, both essential components of the immune system, are classified as inflammatory cells that accumulate at sites of inflammation. Neutrophils are primarily involved in acute inflammation, while lymphocytes play a central role in chronic inflammation. Consequently, the balance between these two cell types, represented by the NLR, serves as a marker not only of the immune status but also of systemic inflammation. An elevated NLR reflects a heightened inflammatory state and has been associated with advanced malignancy and poor prognosis [3,9].

A recent meta-analysis by Wei et al. demonstrated that a high preoperative NLR is significantly associated with worse overall and progression-free survival in gastric cancer patients receiving neoadjuvant chemotherapy (HR for OS = 1.76; 95 % CI, 1.22-2.54; p = 0.003) [11]. Additionally, Zhang et al. conducted a systematic review and meta‑analysis showing that elevated NLR correlates with poor overall survival in gastric cancer patients treated with immune checkpoint inhibitors (pooled HR = 1.98; 95 % CI, 1.67-2.35; p < 0.001) [12].

Despite the growing interest in both NETs and NLR, studies exploring their interplay in gastrointestinal cancers remain limited. A recent mechanistic study showed that in gastric cancer, hypoxic tumor microenvironments stimulate NET formation via the HMGB1/TLR4/p38 MAPK pathway, and that elevated NET levels are associated with poorer prognosis [13]. Furthermore, comprehensive reviews have summarized how NETs contribute to tumor progression across gastrointestinal malignancies, including gastric and colorectal cancers, through mechanisms such as immune evasion, metastasis promotion, and remodeling of the tumor microenvironment [14,15].

These observations align with our findings of a significant correlation between systemic NLR and intratumoral NET density. They support the notion that NLR, a simple and cost-effective systemic marker, may reflect local immune processes such as NET activity, which contribute to gastric cancer progression. Our data thus bolster the clinical utility of NLR as a surrogate for tumor-associated NET formation.

However, this study has several limitations that must be acknowledged. It was retrospective in nature and included a relatively small number of patients with heterogeneous clinical characteristics. Additionally, multivariate analyses adjusting for potential confounding factors such as tumor stage, histological type, or treatment variables were not conducted, which may have influenced the observed associations between NLR, NETs, and survival outcomes. The study also lacked functional analyses to directly elucidate the mechanistic pathways involved in NET formation and NLR elevation. Future studies should include larger cohorts, prospective designs, and integrated molecular analyses to further validate these findings and explore the therapeutic potential of targeting NET-related pathways.

## Conclusions

To our knowledge, this is the first study to identify a relationship between circulating NLR and intratumoral NETs in gastric cancer. These observations support the potential utility of NLR as a surrogate marker for tumor-associated NET formation. Further investigation is warranted to determine whether targeting NET-related pathways holds therapeutic promise in clinical oncology.
